# Acute Renal Vein Thrombosis Following COVID-19 in a Lupus Patient: A Case Report and Review of the Literature

**DOI:** 10.3390/life13061252

**Published:** 2023-05-25

**Authors:** Dimitra Petrou, Aggeliki Sardeli, Panayiotis Vlachoyiannopoulos, Ornella Moschovaki-Zeiger, Sophia Lionaki

**Affiliations:** 1 Department of Nephrology, Attikon Hospital, National and Kapodistrian University of Athens, 12462 Athens, Greece; 2 Department of Pathophysiology, School of Medicine, National and Kapodistrian University of Athens, 11527 Athens, Greece; 3 Department of Radiology, Attikon Hospital, National and Kapodistrian University of Athens, 12462 Athens, Greece

**Keywords:** COVID-19, SARS-CoV-2, renal infarction, renal thrombosis, systemic lupus, erythematosus

## Abstract

**Purpose**: The association between COVID-19 and hypercoagulability is well established. This is a case of a patient with systemic lupus erythematosus (SLE) who developed unilateral renal vein thrombosis following COVID-19, the third case described in the international literature so far. **Methods:** Clinical, laboratory characteristics and outcomes of the patient were described in detail. Literature review was performed on MEDLINE database via Pubmed. Search items included COVID-19, renal infarction, and renal thrombosis. A total of fifty-three cases were located. Of these, only two patients had renal vein thrombosis but none of them carried a diagnosis of SLE. However, six cases have been published so far involving SLE patients in whom thromboembolic events developed following COVID-19, but none of them experienced renal vein thrombosis. **Conclusion:** The present case adds a new piece to the emerging puzzle of COVID-19 associated hypercoagulability, especially among patients with autoimmune diseases.

## 1. Introduction

Severe acute respiratory syndrome coronavirus 2 (SARS-CoV-2) was identified as the cause of a cluster of pneumonia cases in China in 2019 [1]. Since then, coronavirus disease 2019 (COVID-19) has become a pandemic and data is constantly emerging.COVID-19 is strongly associated with hypercoagulability and a high rate of thrombosis, although its pathogenesis is still incompletely understood [2]. We report the first case of renalvein thrombosis following COVID-19 diagnosis in an SLE patient. Through our literature review, we aim to enlighten the association between COVID-19, hypercoagulability, and the kidney, as well as COVID-19, hypercoagulability, and SLE.

## 2. Case Description

A 54-year-old female was admitted to hospital because of back pain expanding in the left abdomen during the previous three days along with low-grade fever for a few hours.

Her past medical history was significant for SLE, hypertension, and dyslipidemia. The patient carried the diagnosis of SLE for 12 years. Upon diagnosis she had arthritis, fever, and leucopenia. Subsequently, she had two episodes of renal involvement, membranous lupus nephropathy, and lupus podocytopathy, both of which were confirmed by histopathology. She remained on remission on hydroxychloroquine, cyclosporine, and low dose methylprednisolone. Six months prior to admission, she had been vaccinated against SARS-CoV-2 (first dose of BioNTech, Pfizer), but a month prior to presentation, she experienced COVID-19 with fever and productive cough for three consecutive days. The SARS-CoV-2 polymerase chain reaction (PCR) was positive on the nasopharyngeal swab, and COVID-19 was diagnosed with no need for οxygen therapy, antiviral treatment, or hospitalization. No differences in arterial blood pressure were noticed before and after COVID-19.

**Figure 1:**
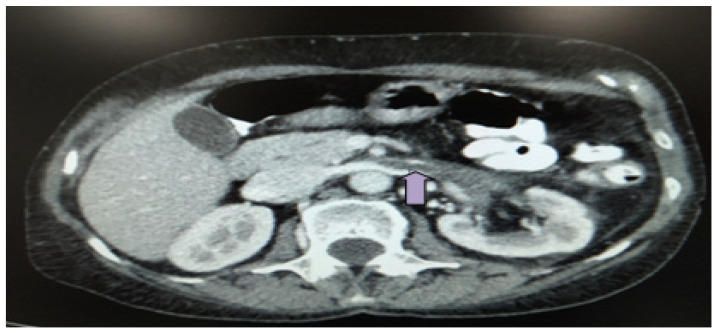
Abdominal CT scan/angiography upon presentation documenting acute thrombosis of the left renal (arrow) and ovarian vein with subsequent development of collateral capsular venous network (axial level).

At presentation, she was afebrile and normotensive. Clinical examination revealed tenderness in the deep palpation of the left side in the absence of a positive Giordano sign. Νo peripheral edema or macroscopic hematuria was noted. Laboratory exams revealed a serum creatinine of 1.1 mg/dL, at baseline, corresponding to an eGFR of 60 mL/min/1.73 m^2^ [3], and increased inflammatory markers (WBC: 10.100 × 10^3^/μL, Neut: 8.500 × 10^3^/μL, CRP: 85 mg/dL) and microscopic hematuria (RBC 10–20/hpf). Renal function was at baseline with no change of proteinuria (Table 1). An abdominal ultrasound showed no abnormalities but an abdominal computed tomography (CT) scan/angiography revealed acute thrombosis in the left renal and ovarian vein with subsequent development of collateral capsular venous network. Ureteric wall thickening of the proximal 2/3 with ureteric varicosities was also identified. No atheroma has been detected, which could serve as a favorable factor of renal vein thrombosis formation upon it (Figure 1).

**Figure 2:**
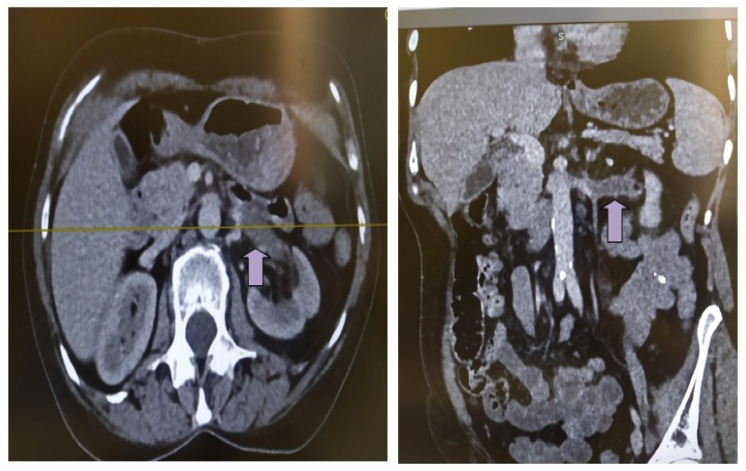
Abdominal CT scan/angiography during follow-up (1 month after renal vein thrombosis diagnosis) revealed renal vein re-tunneling (arrow) (axial and frontal level).

Co-existence of nephrotic syndrome, malignancy, pancreatitis, secondary antiphospholipid syndrome, thrombophilia, and myeloproliferative neoplasms were ruled out with appropriate investigations, according to the workup for visceral thrombosis. Pulmonary embolism was ruled out with a CT pulmonary angiogram. The patient was treated with anticoagulant therapy (tinzaparin 175 IU/kg/24 h) with significant clinical and laboratory improvement (Table 1). In the absence of any other etiology for visceral thrombosis, the association of thrombosis with the recent COVID-19 disease was considered more likely. Τinzaparin administration was interrupted after 1 month and was immediately replaced by apixaban 5 mg b.i.d. During the follow up, the patient demonstrated complete resolution of symptoms anda decrease in inflammatory markers, and microscopic hematuria resolved. Renal vein re-tunneling was shown on subsequent screening (Figure 2).

## 3. Methods

Two different literature reviews were performed on the MEDLINE database via Pubmed in order to identify documented cases of renal infarcts or thrombosis following COVID-19 (49 articles—53 cases) and documented thromboembolic events on SLE patients following COVID-19 (6 articles—6 cases).

The first literature review was performed on MEDLINE database via Pubmed on October 2022 in order to identify documented cases with renal infarcts or thrombosis following COVID-19. The study entry criteria were studies of any type that concerned exclusively human adults with COVID-19 or asymptomatic people with a positive molecular test against SARS-CoV-2 who had a documented renal thrombosis or infarction following COVID-19. No restrictions were imposed on the language of publication or country where the studies were conducted. The exclusion criteria included studies involving animals, people younger than 18 years old, or without documented results of interest. The search algorithm was conducted using the following terms in order to locate all relevant records: [(coronavirus or COVID or COVID-19 or SARS-CoV-2) AND (renal or kidney) and (thrombosis OR infarction)].

The second literature review was conducted in order to identify documented thromboembolic events on SLE patients following COVID-19 diagnosis. The last search was carried out on the 31 October 2022 as well. The study entry criteria were studies of any type that concerned, exclusively, human adults with SLE or COVID-19, or who were asymptomatic with a positive molecular test against SARS-CoV-2 and a documented thromboembolic event following COVID-19. No restrictions were imposed on the language of publication or the country where the studies were conducted. Exclusion criteria included studies involving animals, people younger than 18 years old, or without documented results of interest. The search algorithm was conducted using the following terms in order to locate all relevant records: [(lupus or systemic lupus erythematosus or SLE) and (coronavirus or COVID or COVID-19 or SARS-CoV-2) and (thrombosis or infarction or thromboembolic)].

## 4. Results

The first literature review was carried out on the 31 October 2022 and initially identified 612 relevant published articles. Enrolled articles were obtained by searching references in the articles found in the primary search. A total of 49 relevant studies (case reports and case series) were identified after adjusting for duplicates and exclusion of articles based on title and abstract or after full-text examination. These 49 published articles included 53 cases in total (Table 2). Fifty one cases were shown to have renal artery infarction following COVID-19, whereas two cases reported renal vein thrombosis following COVID-19. None of them had a medical history or received a new diagnosis of SLE. Only 8% of patients had a history of chronic kidney disease, whereas 2% had a history of atrial fibrillation. Regarding gender, 72% of them were males and 28% were females. The average age of patients was 53 years old (average age for men was 53 years old, and average age for women was 54 years old). Τhe vast majority involved native kidneys (81%),while the rest concerned transplanted kidneys (19%).Unilateral infarctions were documented in the majority of cases (77%). Bilateral infarctions were revealed inalmost a quarter of the cases (23%), concerning 75% male and 25% female patients. No article detected positive antiphospholipid antibodies (LA, anti-β2-GPI, Acl). Finally, the majority of the studies took place in the United States (40%) followed by Asia (26%), Europe (20%), South America (10%), and Africa (4%).

The second literature review initially identified 103 relevant published articles. Enrolled articles were obtained by searching references in the articles found in the primary search. A total of six relevant reports were identified after adjusting for duplicates and the exclusion of articles based on title and abstract or after full-text examination. The six published articles included in total six cases (Table 3). All of the cases documented a thromboembolic event in SLE patients following COVID-19. Different types of thromboembolic events were documented (one case with deep vein thrombosis, one case with concurrent deep vein thrombosis and pulmonary embolism, one spleen infarct, one small intestinal microvascular thrombosis, and one acute recurrent acute upper limb ischemia). Although, no renal vein thrombosis was identified as in our case analyzed above. Τwo of the cases (33.3%) described documented a thromboembolic event in the setting of a new-onset SLE diagnosis following COVID-19, whereas the other four cases (66.6%) had a prior history of SLE with an average duration of 5 years. Only one case (16%) had a prior history of antiphospholipid syndrome secondary to SLE, whereas another patient (16%) had a history of prior positive aPL in the absence of APS. Regarding gender, all of the patients were female, with an average age of 29 years old. Finally, the majority of the studies took place in the United States (50%) followed by Europe (33%) and Asia (16%).

## 5. Discussion

COVID-19 has been strongly associated with hypercoagulability and a high rate of thrombosis, although the related pathogenesis is still incompletely understood. Hypercoagulability can be thought of in terms of Virchow’s triad, affecting all three branches. First, it has been established that viral particles directlyaffect endothelial cells, leading to endothelial injury. Complement activation has also been implicated and is a subject of ongoing research and potential therapeutic targets. However, stasis due to patient immobilization and self-isolation or the need for hospitalization plays a major role, as well as hyperviscosity due to a number of changes in circulating prothrombotic factors (elevated factor VIII, fibrinogen, circulating prothrombotic microparticles, and neutrophil extracellular traps (NETs) [2].

The spectrum of thromboembolic manifestations is broad and varies widely among different individuals and clinical studies. The vast majority of the cases experience venous thromboembolism (VTE), including extensive deep vein thrombosis (DVT) and pulmonary embolism (PE) [2]. Microvascular lung and renal thrombosis have been identified in large autopsy series. Several cases with arterial thrombosis have also been documented, including stroke, myocardial infarction, and limb ischemia. Arterial thrombosis is strongly associated with increased mortality [59].

The aim of the current literature research was to identify the spectrum of renal infarcts or thrombosis following COVID-19. A total of 53 patients were identified with documented thrombosis following COVID-19. The vast majority (96%) of them involved renal artery thrombosis (RAT), whereas renal vein thrombosis (RVT) was documented only in two cases. As a result, we consider our patient as the third documented case worldwide demonstrating the even broader spectrum of COVID-19 kidney hypercoagulability interaction.

Renal artery thrombosis refers to the formation of a blood clot in one or both renal arteries. Early diagnosis and anticoagulation therapy is pivotal to preventing renal impairment. The most common cause is thromboembolism originating in the heart, followed by traumatic injury, thromboembolism originating in aorta, cancer, pregnancy, nephritic syndrome, SLE, infective endocarditis, renovascular hypertension, and genetic conditions [60,61,62]. It is important to emphasize that only one case (2%) in the literature review had a prior history of atrial fibrillation, whereas none of them were a lupus patient. Furthermore, renal vein thrombosis (RVT) is a relatively rare condition occurring in a clinical setting, such as nephrotic syndrome, renal malignancy, trauma, inherited procoagulant disorders, and severe hypovolemia (especially in infants) [62]. Males are affected more often than females. Bilateral involvement is described in almost two thirds of patients. In cases of unilateral thrombosis the left renal vein is affected more commonly than the right [63]. Renal vein thrombosis is more often chronic, as a result of nephrotic syndrome, and has an insidious onset and may produce no symptoms. Acute RVT typically presents with flank pain and microscopic or gross hematuria, and it may be a result of trauma or a hypercoagulable state. Bilateral RVT can present with acute kidney injury (AKI) [64]. Treatment includes therapeutic anticoagulation (unless contraindicated), initially with unfractionated or heparin of a low-molecularweight, and then warfarin. No robust data exist on the use of DOACs for RVT so far [65]. Patients with RVT and AKI are candidates for dissolution or removal of the thrombus with thrombolytic therapy, with or without thrombectomy [63,64]. All three patients detected in international literature with RVT following COVID-19 had a unilateral involvement, and the left renal vein was affected in 66% of them. Acute renal vein thrombosis (RVT) can result in CKD, especially when both renal veins are affected or when insidious onset results in delayed diagnosis and treatment. None of these three cases resulted in CKD due to unilateral involvement and early diagnosis and treatment with anticogulation therapy. Although men are usually more affected [63], two of the three cases were females. All of them had symptomatic acute RVT in the absence of AKI, which resolved with anticoagulation therapy. One patient died as a result of COVID-19 respiratory complications.

Patients with autoimmune diseases tend to have a higher incidence of viral infections in general, likely due to a combination of immune dysfunction, immunosuppressive therapy, and co-morbidities [66]. Corticosteroids, particularly at increased doses, reduce innate, cell-mediated, and humoral immune responses to infection and increase the risk of opportunistic infections. As a result, they may increase the risk of contracting COVID-19. Advanced age, hypertension, chronic pulmonary, and kidney disease, obesity, and diabetes are common comorbidities of patients with rheumatic diseases [67]. However, they are also risk factors for severe COVID-19. Lupus anticoagulant antibodies (LA) positivity in COVID-19 has been described in several articles. Approximately 30% of SLE patients are positive for LA. However, in order to diagnose antiphospholipid syndrome (APS), both clinical as well as laboratory criteria must be met [66]. Antiphospholipid antibodies (LA, anti-β2-GPI, Acl) may be transient elevated in several conditions (particularly infections and critical illness). As a result, persistent elevation for at least 12 weeks must be documented in order for aPL to be deemed positive. Several studies have documented the presence of positive aPL in COVID-19 patients. However, their clinical significance in COVID-19 patients remains undefined, since there is no concrete data if there is indeed a higher incidence of thromboembolic events in clinical practice [68].

Data regarding SLE and COVID-19, in particular, remain limited and scarce [69]. Since the SLE patient in this report demonstrated a major thrombotic event following COVID-19, we conducted a second literature review in order to identify documented thromboembolic events in SLE patients following COVID-19. Overall, six cases were identified, including different types of thromboembolic events (one DVT, one concurrent DVT and PE, one spleen infarct, one small intestinal microvascular thrombosis, and one acute treatment-resistant acute upper limb ischemia). No RVT case has been described up until now. As a result, our case report adds another piece in the COVID-19 SLE emerging puzzle. Two of the identified cases reported a new-onset SLE diagnosis following COVID-19. One SLE individual had a prior history of secondary APS, under treatment with warfarin, without new thrombotic manifestations for 10 years prior to COVID-19. Another case with a prior history of positive aPL in the absence of APS experienced a treatment-refractory artery thrombosis, leading to limb amputation following COVID-19 diagnosis. “Second hit” hypothesis supports that COVID-19 may serve as the precipitating factor of secondary APS in an immune modified substrate in individuals with autoimmune diseases, such as SLE patients.

In conclusion, the present case is the third RVT documentation following COVID-19 and the seventh case of COVID-19 associated hypecoagulability in the setting of SLE. Therefore, it adds a new piece in the emerging landscape of COVID-19 associated hypercoagulability, especially among patients with autoimmune diseases.Increased vigilance among clinicians is required in the management of patients with lupus, particularly during the COVID-19 pandemic.

## Figures and Tables

**Table 1:** Laboratory findings before COVID-19, after COVID-19, upon presentation with renal vein thrombosis, and during follow-up period.

	Before COVID-19 (2 Months Prior)	After COVID-19 (20 Days Prior)	Upon Presentation	Follow-Up (3 Months After)
WBC (10^3^/μL)	9.700	9900	10.100	6.500
Neut (10^3^/μL)			8.383	
Hgb(gr/dL)	13	13.5	12	13.5
PLT (10^3^/μL)	200.000	234.000	152.000	248.000
CRP (mg/dL)	4	9	85	5
Serum Urea (mg/dL)	61	72	56	270
Serum Creat (mg/dL)	1.1	1.2	1	1.1
eGFR (mL/min/1.73 m^2^)	52	47	58	52
Na^+^ (mmol/L)	143	143	138	142
K^+^ (mmol/L)	4.1	4.2	4.4	4.4
Ca^2+^ (mg/dL)	9.2	9.2	8.8	9.3
alb (g/dL)	3.3	3.4	3.5	3.6
Urine	RBC 2–3/hpf	RBC 1–2/hpf	RBC 10–20/hpf,	RBC 2–3/hpf
	WBC 0–2/hpf	WBC 1–2/hpf	WBC 0–2/hpf	WBC 1–2/hpf
Proteinuria (mg/24 h)	650	NA	850	900

**Table 2:** Main characteristics of the 49 articles/53 cases identified in the first literature review in order to recognize all documented cases with renal infarcts or thrombosis following COVID-19 diagnosis in the general population.

Author- Continent	S	Infarction Type (A/V)	Native/ Allograft	Gender -Age	SLE	CKD	AF	Unilateral /Bilateral
Kudose et al [4], USA	1	A	Na	M50	N	N	N	U
Satturwar et al. [5], USA	4	A	Na	M52,M53, F48, F49	N	N	N	1B 3U
Akin et al. [6], AS	1	A	Na	M48	N	N	N	U
Takamatsu et al. [7], USA	1	A	Na	M71	N	N	N	B
Xu et al. [8], USA	1	A	Allo	M46	N	Y	N	U
Añazco et al [9], SA	1	A	Na	F41	N	N	N	B
Singh et al. [10], AS	1	A	Na	M32	N	N	N	U
El Shamy et al. [11], USA	1	A	Na	F60	N	N	Y	B
Acharya et al. [12], USA	1	A	Na	F77	N	N	N	U
Ren et al. [13], AS	1	A	Na	F53	N	N	N	U
Daniel et al [14], USA	1	A	Allo	M29	N	Y	N	U
Mukherjee et al. [15], US	1	A	Na	M71	N	N	N	U
Bonello et al. [16], EU	1	A	Na	M47	N	N	N	B
Ammous et al. [17], USA	1	A	Na	M62	N	N	N	U
Belfort et al. [18], USA	1	A	Na	M28	N	N	N	U
Post et al. [19], EU	2	A A	Allo Na	M62 M58	N N	Y N	N N	U U
Mantica et al. [20], EU	1	A	Na	F67	N	N	N	U
Tascón et al. [21], EU	1	A	Na	M56	N	N	N	U
Mavraganis et al. [22], EU	1	A	Na	M64	N	N	N	U
Jana et al. [23], USA	1	A	Na	M37	N	N	N	B
Varmer et al. [24], USA	1	A	Na	M46	N	N	N	U
Rametke et al. [25], AS	1	A	Na	M66	N	N	N	B
Tantisattamo et al. [26], USA	1	A	Allo	F33	N	N	N	U
Dimosiari et al. [27], EU	1	A	Na	M75	N	N	N	B
Sethi et al. [28], AS	1	A	Na	M62	N	N	N	U
Ramanthan et al. [29], USA	1	A	Na	M54	N	N	N	U
Huang et al. [30], AS	1	A	Na	M62	N	N	N	U
Varma et al. [31], AS	1	A	Allo	M47	N	N	N	U
Berrichi et al. [32], AFR	1	A	Na	M45	N	N	N	B
Jentzsch et al. [33], USA	1	A	Na	F28	N	N	N	U
Farias et al. [34], SA	1	A	Na	M37	N	N	N	U
Mocerino et al. [35], USA	1	A	Na	F69	N	N	N	U
Veterano et al. [36], EU	1	A	Allo	F56	N	Y	N	U
Al-Mashdali et al. [37], AS	1	A	Na	M43	N	N	N	U
Kenizou et al. [38], EU	1	A	Na	M78	N	N	N	U
Imoto et al. [39], AS	1	A	Na	M64	N	N	N	B
Fluss et al. [40], USA	1	A	Na	M60	N	N	N	U
Horiguchi et al. [41], AS	1	A	Na	M58	N	N	N	U
Kurien et al. [42], AS	1	A	Na	M32	N	N	N	B
Belfiore et al. [43], EU	1	A	Na	F76	N	N	N	B
Al Mousa et al. [44], AS	1	A	Na	M31	N	N	N	U
Espinosa et al. [45], USA	1	A	Na	F21	N	N	N	U
Jain et al. [46], AS	1	A	Na	M62	N	N	N	U
Murray et al. [47], SA	1	A	Na	M25	N	N	N	U
Brem et al. [48], AFR	1	A	Na	M59	N	N	N	U
Philipponnet et al. [49], EU	1	A	Na	M52	N	Y	N	U
Mancini et al. [50], EU	1	A	Na	M43	N	N	N	U
Asleson et al. [51], USA	1	V	Na	F44	N	N	N	U
Mirfakhraee et al. [52], AS	1	V	Na	M74	N	N	N	U

(Abbreviations:AS, Asia; EU, Europe; AFR, Africa; SA, South America; S, Sample size; A, Arterial; V, Venous; Na, Native; Allo, Allograft; M, Male; F, Female; Y, Yes; N, No; U, Unilateral; B, Bilateral).

**Table 3:** Main characteristics of the six cases spotted in the second literature review identifying thromboembolic events in SLE patients following COVID-19 diagnosis.

Author- Continent	S	Gender -Age	SLE Duration	Prior History of APS	System Involvement	Immunological Factors	SLE Medications	Type of Coagulopathy
Kincaid et al. [53], USA	1	F43	10 years	Y	Neuropsychiatric	NA	HCQ, MMF, Oral CS, Warfarin	Acute ischemic cerebral stroke
Tuğsal et al. [54], AS	1	F20	New-onset	N	Respiratory, pleural effusions, pericardial effusion	Positive ANA, anti-ds DNA	NA	Spleen infarct
Aguirre-Alastuey et al. [55], EU	1	F22	2 years	N	Arthritis, thrombocytopenia	Positive ANA, anti-ds DNA, aPL	HCQ MTX Oral CS aspirine	DVT, PE, Secondary APS
Nespola et al. [56], EU	1	F47	NA	N	NA	Positive LA	Oral CS	Acute treatment-resistant recuurent upper limb ischemia
Plotz et al. [57], USA	1	F27	4 years	N	Haematological, Cutaneous, arthritis	Positive ANA, anti-ds DNA, anti-Ro, anti-La, Low c3,c4	NA	Smallintestinalmicrovascularthrombosis
Mantovani et al. [58], USA	1	F18	New-onset	N	Haematological, renal, respiratory, pleural effusions, pericardial effusion, and tamponade	NA	NA	DVT, Secondary APS

(Abbreviations: AS, Asia; EU, Europe; AFR, Africa; SA, South America; S, Sample size; M, Male; F, Female; Y, Yes; N, No; NA, Not available; HCQ, Hydroxychloroquine; CS, Corticosteroids; MTX, Methotrexate; APS, Antipshospholipid syndrome; DVT, Deep veinthrombosis; PE, Pulmonary embolism.)

## Data Availability

The data that support the findings of this study are available from the corresponding author, upon reasonable request.
